# Supplementary material for: Smartwatch-based detection of loss of pulse
Source: PLoS One. 2026 Feb 27;21(2):e0342064. doi: 10.1371/journal.pone.0342064 (PMC12948053; doi:10.1371/journal.pone.0342064)
Supplement: Attachment — Submitted filename: PLOS___LossOfPulse_response.pdf [file pone.0342064.s001.pdf]

PLOS ONE, Editorial office,
August 4, 2025

Please find enclosed our response to the comments and changes:

**Manuscript Number:**	**PONE-D-25-29278**
**Title:**	**Smartwatch-Based Detection of Loss of Pulse**

We would like to thank the reviewers for their useful comments on our paper. We have provided point-by-point responses to their comments and questions. We have also made several changes to the paper in response to their concerns.

In the updated manuscript, all the changes are marked in **Blue**. The replies to the itemized comments are included where the responses are colored **Green**, and the corresponding changes to the manuscript are pasted and colored **Blue**.

Sincerely,

Authors.

Comments And Responses:

Reviewer 1 comments/questions: (1):
Classification Labels and Evaluation Metrics

Response:
We thank the reviewer for this question. Our task is a three-class classification problem (pulse loss, normal operation, idle). However, for reporting sensitivity and specificity, we combined normal operation and idle into a single class (label 0) and treated pulse loss as the positive class (label 1), effectively framing it as a binary problem for those metrics.

In response to the reviewer's suggestion, we have now included Macro-averaged F1 Score across all three classes in the revised tables.

We have updated Figure 4 and its caption to include all classes, following the reviewer's suggestion.

Reviewer 1 comments/questions: (2):
Terminology: "Deep Learning" vs. "Neural Network"

We thank the reviewer for this careful review. We have revised the script to refer our model as dual-stream CNN-LSTM model as per suggestion.

Reviewer 1 comments/questions: (3):
Proposed Method Identification

The deep learning architecture, dual-stream CNN-LSTM, was used as our method. We made this distinction in tables and throughtout the manuscript.

Reviewer 1 comments/questions: (4):
Feature Dimensions and Signal Processing

Yes, for models relying on feature extraction, we computed features separately for each of the three IMU axes and concatenated them to form the final input feature vector. This approach preserves axis-specific information and enables the classifiers to leverage the full sensor data. We have added the text below to the revisited manuscript while explaining the number of extracted features.

Features were extracted independently from each channel, one PPG and three IMU axes, and then concatenated as input to the classifiers. Overall, we used ten features from the PPG channel and 21 features from the IMU signals (7 features per axis $\times$ 3 axes).

For our deep learning model, which performs end-to-end learning without explicit feature extraction, we fed the raw IMU signals directly without additional preprocessing such as filtering or normalization.

Regarding signal synchronization, the smartwatch hardware provides temporally aligned PPG and IMU signals; therefore, no additional resampling or time-alignment was applied.

Reviewer 1 comments/questions: (5):
Ambiguity in "IMU" Definition

We thank the reviewer for this suggestion. We only used 3-axis acceloremeter. We made this clear in the revision by changing the paragraph below in Section 3.4.

For the inertial measurements, we used the raw signals from the 3-axis accelerometers, segmented in the same manner as the PPG signals without applying any filtering or normalization.

Reviewer 1 comments/questions: (6):
Cross-Validation and Subject Separation

We confirm that strict Leave-One-Subject-Out (LOSO) cross-validation was applied for both traditional classifiers and the deep learning model. For the deep learning model, LOSO was also strictly enforced. Data from the test subject was fully excluded during training, maintaining the integrity of the cross-validation.

Reviewer 1 comments/questions: (7):
PPG-DaLiA Evaluation and Ground Truth

We used the PPG-DaLiA dataset to evaluate our model's false alarm rate on real-world data containing only normal usage, where no pulse loss or non-usage labels are available. Since the dataset exclusively reflects normal conditions, the model ideally should not trigger alarms, providing a practical test of its precision. This evaluation approach follows protocols from prior work [1], which we have adapted for consistency.

Reviewer 1 comments/questions: (8):
Comparison with Existing Methods

We thank the reviewer for this important question. We found only one relevant study [1] on pulse loss detection using smartwatches. However, a direct comparison is challenging due to significant differences in hardware: their approach employs multiple PPG channels along with integrated audio and haptic notifications. In contrast, our method targets a more limited hardware setup to ensure broader applicability. Given these differences, an exact comparison is not feasible. Nevertheless, we welcome any specific suggestions or datasets from the reviewer and would be happy to conduct additional experiments accordingly.

Reviewer 2 comments/questions: (1):
The smartwatch (Samsung Gear S3 Frontier) characteristics.

We thank the reviewer for the suggestion. Detailed specifications such as vendor, resolution, and maximum range of the Samsung Gear S3 Frontier sensors are not publicly available. Since our method is designed as a general solution rather than hardware-specific, we validated its robustness on the PPG-DaLiA dataset, which only includes normal usage, and observed a significantly low false detection rate. This demonstrates the method's effectiveness beyond a specific device setup.

Reviewer 2 comments/questions: (2):
"An example setup of the data collection is shown in Figure 1" in page 4.

We thank the reviewer for this careful review. We have removed the initial reference to Figure 1 and retained only the subsequent one for clarity.

Reviewer 2 comments/questions: (3):
The reference to Figure 3 in page 5 is not clear. I am not sure if it is really to Figure 3 or Figure 2. Check it.

We thank the reviewer for pointing this out. We have corrected the reference to clearly point to the appropriate figures. Additionally, we have ensured that all figures, including Figures 2 and 3, are properly referenced and explained in the revised manuscript for clarity as in below.

Examples of the event definition for the baseline (2a), the pulse loss (2b and 3a), and the return of blood flow (3a) cases are given in Figures 2 and 3.

Reviewer 2 comments/questions: (4):
The architecture of the employed neural network model.

We thank the reviewer for this question. Following the suggestion, we have explicitly named our method "dual-stream CNN-LSTM" and provided this reference consistently in the tables. Additionally, a detailed description of the model architecture is included in Section 3.5.1 of the manuscript. We have also included the total number of parameters of our model, as per the reviewer's suggestion.

... The overall architecture has approximately 500k parameters. We also give a schematic illustration of our architecture in Figure 4.

Reviewer 2 comments/questions: (5):
Table 4 (in page 11) should be placed after (or closer) to its reference (page 13).

We thank the reviewer for this suggestion. We moved Table 4 closer to its first mention in the revised manuscript to improve readability and flow.

Reviewer 2 comments/questions: (6):
The sentence in page 15 "Second, when the subjects wore ..." seems to be incomplete.

We thank the reviewer for this careful review. We have removed the word "when" at the beginning of the sentence to improve clarity.

References

[1] K. Shah, A. Wang, Y. Chen, J. Munjal, S. Chhabra, A. Stange, E. Wei, T. Phan, T. Giest, B. Hawkins, D. Puppala, E. Silver, L. Cai, S. Rajagopalan, E. Shi, Y.-L. Lee, M. Wimmer, P. Rudrapatna, T. Rea, S. Yuen, A. Pathak, S. Patel, M. Malhotra, M. Stogaitis, J. Phan, B. Patel, A. Vasquez, C. Fox, A. Connell, J. Taylor, J. Shreibati, D. Miller, D. McDuff, P. Kohli, T. Gadh, and J. Sunshine, "Automated loss of pulse detection on a consumer smartwatch," *Nature*, pp. 1–3, Feb. 2025. [Online]. Available: https://www.nature.com/articles/s41586-025-08810-9
