# Supplementary material for: Smartwatch-based detection of loss of pulse
Source: PLoS One. 2026 Feb 27;21(2):e0342064. doi: 10.1371/journal.pone.0342064 (PMC12948053; doi:10.1371/journal.pone.0342064)
Supplement: Attachment — Submitted filename: Plos_response_to_the_reviewers.pdf [file pone.0342064.s002.pdf]

PLOS ONE, Editorial office,
November 22, 2025

Please find enclosed our response to the comments and changes:

**Manuscript Number:**	**PONE-D-25-29278**
**Title:**	**Smartwatch-Based Detection of Loss of Pulse**

We would like to thank the reviewers for their useful comments on our paper. We have provided point-by-point responses to their comments and questions. We have also made several changes to the paper in response to their concerns.

In the updated manuscript, all the changes are marked in **Blue**. The replies to the itemized comments are included where the responses are colored **Green**, and the corresponding changes to the manuscript are pasted and colored **Blue**.

Sincerely,

Authors.

Comments And Responses:

Reviewer 1 comments/questions: (1):
I would encourage the authors to discuss and compare their approach with other recent work on smartwatch-based loss of pulse detection. For instance, Shah et al. (NShah, K., Wang, A., Chen, Y. et al. Automated loss of pulse detection on a consumer smartwatch. Nature 642, 174–181 (2025). https://doi.org/10.1038/s41586-025-08810-9) presented an automated loss of pulse detection system implemented on a consumer smartwatch, using a multi-stage pipeline with PPG, motion data, and a lightweight convolutional neural network trained on 528 engineered features. Given that your work also focuses on smartwatch-based pulse loss detection with machine learning, it would strengthen the manuscript to position your framework in relation to this paper, highlighting methodological similarities (e.g., multimodal PPG + motion sensing, simulation of pulse loss via occlusion) as well as differences (e.g., dataset openness, model architectures, evaluation settings).

Response:
We thank the reviewer for the suggestion. We agree that positioning our work in relation to Shah et al. (Nature, 2025) strengthens the manuscript. We have updated Section 2 (Related Work) to acknowledge their use of arterial occlusion while highlighting our unique contributions. Furthermore, we have added a paragraph to Section 5 (Discussion) to clarify why a direct algorithmic comparison was not feasible due to the proprietary, hardware-specific nature of their approach.

Changes to Section 2: Related Work

... In a recent development, Google introduced the "Pixel Watch 3", featuring a loss of pulse detection system detailed by Shah et al [1]. Their work demonstrates the viability of using a commercial smartwatch for this purpose, employing a multi-stage pipeline that processes PPG and motion data through a lightweight convolutional neural network trained on 528 engineered features. Notably, Shah et al. utilized arterial occlusion to simulate pulse loss, validating the relevance of the occlusion methodology used in our own study. This approach is crucial for realism, as it allows for data collection from healthy, conscious participants, avoiding the confounding physiological effects of anesthesia and medication inherent to intra-operative clinical studies.

However, despite these advancements, the underlying datasets remain private, and ground truth is often established via secondary indicators like standard pulse oximetry. Our work addresses these gaps by providing a multimodal dataset. Unlike standard clinical labeling, our use of continuous ultrasound imaging also enables us to visually confirm and precisely annotate the exact millisecond of blood flow cessation in the radial artery, minimizing label noise and ensuring the model is trained on high-fidelity physiological endpoints.

Changes to Section 5: Discussion

... Fourth, regarding the state-of-the-art comparison, we note that while Shah et al. [1] also utilized arterial occlusion to simulate pulse loss, their detection pipeline relies on proprietary, device-specific firmware logic. Specifically, the authors use dynamic adjustment of LED currents and photodiode gain upon initial detection to verify pulselessness. Because our dataset consists of passively recorded signals from a standard consumer smartwatch without such active hardware control, a direct application of their specific multi-stage algorithm to our data was not feasible. Future work could address this by developing hardware-agnostic approximations of these active sensing protocols, or by using programmable research platforms to facilitate direct benchmarking across different devices.

References

[1] K. Shah, A. Wang, Y. Chen, J. Munjal, S. Chhabra, A. Stange, E. Wei, T. Phan, T. Giest, B. Hawkins, D. Puppala, E. Silver, L. Cai, S. Rajagopalan, E. Shi, Y.-L. Lee, M. Wimmer, P. Rudrapatna, T. Rea, S. Yuen, A. Pathak, S. Patel, M. Malhotra, M. Stogaitis, J. Phan, B. Patel, A. Vasquez, C. Fox, A. Connell, J. Taylor, J. Shreibati, D. Miller, D. McDuff, P. Kohli, T. Gadh, and J. Sunshine, "Automated loss of pulse detection on a consumer smartwatch," *Nature*, pp. 1–3, Feb. 2025. [Online]. Available: https://www.nature.com/articles/s41586-025-08810-9
